# Deficiency of *Trex1* leads to spontaneous development of type 1 diabetes

**DOI:** 10.1186/s12986-023-00777-6

**Published:** 2024-01-02

**Authors:** Jiang-Man Zhao, Zhi-Hui Su, Qiu-Ying Han, Miao Wang, Xin Liu, Jing Li, Shao-Yi Huang, Jing Chen, Xiao-Wei Li, Xia-Ying Chen, Zeng-Lin Guo, Shuai Jiang, Jie Pan, Tao Li, Wen Xue, Tao Zhou

**Affiliations:** 1 Nanhu Laboratory, National Center of Biomedical Analysis, Beijing, 100850 China; 2 Beijing Tongren Eye Center, Beijing Tongren Hospital of Capital Medical University, Beijing, 100730 China; 3 Institute of Translational Medicine, School of Medicine, Zhejiang University, Hangzhou, 310029 Zhejiang Province China

**Keywords:** TREX1, Type 1 diabetes, Type I interferon

## Abstract

**Background:**

Type 1 diabetes is believed to be an autoimmune condition, characterized by destruction of insulin-producing cells, due to the detrimental inflammation in pancreas. Growing evidences have indicated the important role of type I interferon in the development of type 1 diabetes.

**Methods:**

*Trex1*-deficient rats were generated by using CRISPR-Cas9. The fasting blood glucose level of rat was measured by a Roche Accuchek blood glucose monitor. The levels of insulin, islet autoantibodies, and interferon-β were measured using enzyme-linked immunosorbent assay. The inflammatory genes were detected by quantitative PCR and RNA-seq. Hematein-eosin staining was used to detect the pathological changes in pancreas, eye and kidney. The pathological features of kidney were also detected by Masson trichrome and periodic acid-Schiff staining. The distribution of islet cells, immune cells or ssDNA in pancreas was analyzed by immunofluorescent staining.

**Results:**

In this study, we established a *Trex1*-deletion Sprague Dawley rat model, and unexpectedly, we found that the *Trex1*^*−/−*^ rats spontaneously develop type 1 diabetes. Similar to human diabetes, the hyperglycemia in rats is accompanied by diabetic complications such as diabetic nephropathy and cataract. Mechanistical investigation revealed the accumulation of ssDNA and the excessive production of proinflammatory cytokines, including IFN-β, in *Trex1* null pancreas. These are likely contributing to the inflammation in pancreas and eventually leading to the decline of pancreatic β cells.

**Conclusions:**

Our study links the DNA-induced chronic inflammation to the pathogenesis of type 1 diabetes, and also provides an animal model for type 1 diabetes studies.

**Supplementary Information:**

The online version contains supplementary material available at 10.1186/s12986-023-00777-6.

## Background

Type 1 diabetes (T1D) is a chronic autoimmune disease characterized by the destruction of pancreatic β-cells, resulting in impaired insulin secretion and high levels of blood glucose. Genetic susceptibility and environmental factors, like virus infections and diet, have been implicated in the development of T1D [1–3]. Currently, it is believed that the destruction of β cells in T1D results from combined actions of both innate and adaptive immune cells [4–6]. And emerging evidences have shown that type I interferon (IFN), as a crucial factor in both innate immunity and adaptive immunity, plays a key role in the development of T1D [7, 8].

Animal models, especially mice and rats, play an essential role in the studies of pathogenesis and treatment of T1D. These models include the spontaneous models like Non-Obese Diabetic (NOD) mice and BioBreeding (BB) rats [9–11], as well as induced models such as streptozotocin (STZ)-induced diabetes model and alloxan-induced diabetes model [12, 13]. Employment of these models in T1D research has provided important insights into the development of this disease. However, a better model that presents autoimmune-related pathogenesis and exhibits more comprehensive disease-associated manifestations is still necessary.

Three Prime Repair Exonuclease 1 (TREX1) is a DNA exonuclease that functions in the degradation of cytosolic DNA [14, 15]. The absence or dysfunction of TREX1 thus leads to the accumulation of cytosolic DNA, which in turn activates the cytosolic DNA sensor, cyclic GMP-AMP synthase (cGAS), and promote the expression of type I IFN and other proinflammatory cytokines through signal transduction cascade [16, 17]. Hence, deficiency of *TREX1* results in sustained activation of cGAS-mediated immune responses which eventually leads to tissue inflammation and damage. Mutations in *TREX1* are associated with the development of several autoimmune diseases, including Aicardi-Goutières Syndrome (AGS) [18], systemic lupus erythematosus (SLE) [19], familial chilblain lupus (FCL) [20], and retinal vasculopathy with cerebral leukodystrophy (RVCL) [21]. In this study, we found that deficiency of *Trex1* in rats leads to spontaneous development of T1D, accompanied by complications such as diabetic cataract and diabetic nephropathy. Mechanistically, *Trex1* deficiency results in the accumulation of single-stranded DNAs (ssDNAs) and excessive production of type I IFN. Thus, our study provides a potential animal model for T1D researches, and may offer new insight into the pathogenesis of T1D.

## Methods

### Rat

Wild-type and *Trex1*^*−/−*^ Sprague Dawley (SD) rats were obtained from Nanjing Biomedical Research Institute of Nanjing University based on CRISPR-Cas9 technology. The guide RNA sequence is as follows: 5′-GTCCACCACACGGGGTGGTT-3′. All rats were housed on a 12 h light/dark cycle with ad libitum access to food and water. All animal experiments were carried out in accordance with the National Institutes of Health Guide for the Care and Use of Laboratory Animals and with the approval of the Institutional Animal Care and Use Committee of the National Center of Biomedical Analysis (IACUC-DWZX—2021-768). WT and *Trex1*^*−/−*^ rats were littermates obtained from heterozygous breeding. Rats were randomly allocated into different groups according to their genotype, and the number of rats were not subjectively reduced or excluded.

### Diabetes assessment and STZ model

**Fig. 1:**
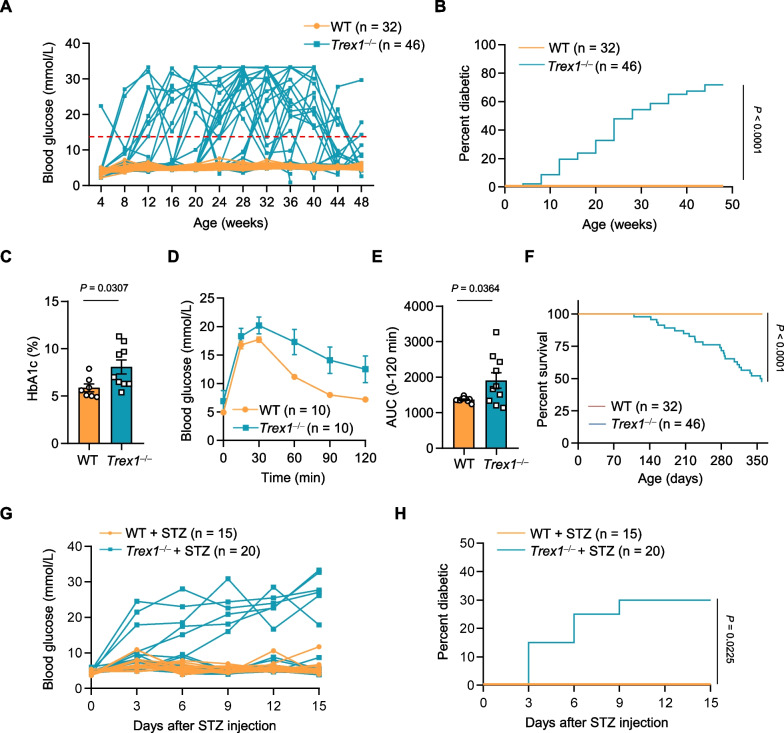
*Trex1*-deficient rats develop spontaneous diabetes. **A** Blood glucose levels of WT (n = 32; female, n = 20; male, n = 12) and *Trex1*^*−/−*^ (n = 46; female, n = 26; male, n = 20) rats were measured monthly from 4 weeks postnatal until 48 weeks, and the individual values of blood glucose were plotted versus ages. **B** Diabetes incidence in WT (n = 32) or *Trex1*^*−/−*^ (n = 46) rats from **A**. **C** Percent HbA1c in WT (n = 7) and *Trex1*^*−/−*^ (n = 9) rats, the detected serum from different weeks of age. **D** The blood glucose changes after challenged with glucose, were measured with time, using WT (n = 10) and *Trex1*^*−/−*^ (n = 10) rats at 12 weeks of age. Glucose was injected intraperitoneally at a dose of 2 g/kg. **E** Quantification of the area under curve (AUC) in **D** for the intraperitoneal glucose tolerance test. **F** Survival curve for WT (n = 32) and *Trex1*^*−/−*^ (n = 46) rats from **A**. **G** Blood glucose was measured periodically from WT + STZ group (n = 15) or *Trex1*^*−/−*^  + STZ group (n = 20) after STZ injection. STZ, streptozotocin, intraperitoneal injection, 30 mg/kg. **H** Diabetes incidence in WT (n = 15) or *Trex1*^*−/−*^ (n = 20) rats after STZ injection. All data are represented as mean ± SEM; Each dot represents one independent biological replicate; Log-rank (Mantel-Cox) test in **B**, **F**, **H**; Unpaired *t* test in **C**; Welch’s *t* test in **E**

Blood glucose levels were measured by a Roche Accuchek blood glucose monitor. Rats were considered as diabetic after two consecutive blood glucose measurements over 13.8 mmol/L. For observation of diabetic phenotypes experiments in Fig. 1A, WT (n = 32; female, n = 20; male, n = 12) and *Trex1*^*−/−*^ (n = 46; female, n = 26; male, n = 20) rats were monitored for one year, with the measurements conducted at a frequency of once a month. For STZ experiments, adult male SD rats (body weight 250–300 g) were fasted for 12–16 h and then treated with a single dose of streptozotocin (STZ, 30 mg/kg of body weight, Sigma-Aldrich, V900890) intraperitoneally. Blood glucose was measured every 3 days post injection.

### Glucose tolerance test and insulin tolerance test

For glucose tolerance test (GTT), rats were fasted for 12–16 h prior to the injection of glucose (2 g/kg of body weight) intraperitoneally. Blood glucose levels were assessed at 0, 15, 30, 60, 90 and 120 min post injection. WT (n = 10; female, n = 4, body weight 259 ± 16 g; male, n = 6, body weight 422 ± 21 g) and *Trex1*^*−/−*^ (n = 10; female, n = 6, body weight 244 ± 15 g; male, n = 4, body weight 377 ± 84 g) rats were used in this experiment. For insulin tolerance test (ITT), rats were fasted for 4 h and received an intraperitoneal injection of insulin (1 U/kg of body weight, MedChemExpress, HY-P0035). Blood glucose levels were evaluated at 0, 15, 30, 60, 90 and 120 min following insulin administration. WT (n = 9; female, n = 3, body weight 287 ± 8 g; male, n = 6, body weight 478 ± 21 g) and *Trex1*^*−/−*^ (n = 10; female, n = 7, body weight 270 ± 22 g; male, n = 3, body weight 433 ± 22 g) rats were detected in this experiment.

### Histological staining

Tissues of pancreas, kidney or eye from rats were fixed with 4% paraformaldehyde for 24–48 h, dehydrated and embedded in paraffin, and subsequently sectioned serially at 4 μm. Paraffin sections were stained using Hematoxylin–Eosin Stain Kit (Solarbio, G1120). PAS and Masson’s trichrome staining of kidney sections were performed by Beijing Xuebang Technology Co., Ltd and Wuhan Servicebio Technology Co., Ltd. All images were obtained using NanoZoomer 2.0 (HAMAMATSU).

### Insulitis scoring

H&E-stained pancreatic sections were scored blindly for islet inflammation. Insulitis scoring was performed as previously described [22]. Scoring was as follows: “non-insulitis” indicates no inflammatory cells infiltration; “peri-insulitis” indicates that inflammatory cells surround around the pancreatic islets and with less than 25% area of islets being impaired; “non-aggressive insulitis” refers to 25–50% area of pancreatic islets infiltrated with inflammatory cells; “aggressive insulitis” refers to almost destroyed islets with or without residual inflammatory cells.

### Immunofluorescence

Paraffin embedded tissue sections were blocked with 5% normal goat serum for 1 h, and then incubated overnight at 4 °C with primary antibodies as follows: anti-Insulin (1:100, Abcam, ab7842), anti-Glucagon (1:150, Cell Signaling Technology, 2760S), anti-CD8 (1:100, Bio-Rad, MCA48GA), anti-CD68 (1:100, Bio-Rad, MCA341GA), anti-DNA (1:20, Millipore, single stranded specific, MAB3299). Secondary antibodies were employed as follows: Alexa Fluor 488 (1:400, Jackson ImmunoResearch or Thermo Fisher Scientific) and Alexa Fluor 594 (1:400, Thermo Fisher Scientific). Nuclei were stained with Hoechst (1:1000, Thermo Fisher Scientific, H3570). Images were acquired via ZEISS LSM 880 confocal microscope, and data collection was performed using ZEN 2.1 SP2 Black version 13.0.2.518 (ZEISS). The statistical analysis of fluorescent images was performed independently and blindly by two individuals.

### RNA extraction and quantitative PCR

Pancreas tissues were lysed through liquid nitrogen grinding rapidly. And total RNA was then isolated using TRI reagent (Sigma, T9424). RNA was reversed into cDNA by Prime Script RT Master Mix (Takara, RR036A). Quantitative PCR (qPCR) was performed with SYBR Green Master Mix (Thermo Fisher Scientific, A25778) on Applied Biosystems QuantStudio 3/5 Real-Time PCR System (Applied Biosystems). Relative expression of tested genes was normalized to housekeeping gene *Gapdh* and control samples through the 2^−ΔΔCt^ method. Primer sequences used in this study are provided in Additional file 1: Table S1.

### RNA-seq

Briefly, total RNA was isolated from the pancreas of WT or *Trex1*^−/−^ rats and the integrity of RNA was determined on an Agilent 2100 bioanalyzer. The transcriptome library was then constructed and sequenced via the Illumina sequencing platform (pair-end 150 bp) of Annoroad Gene Technology (Shanghai, China). Sequencing reads were aligned to RN6 using HISAT2 and differentially expressed genes (DEGs, adjusted P < 0.05 and fold change > 2 or fold change < 0.5) were identified with DESeq2. The up-regulated differentially expressed genes in RNA-seq data were analyzed and fold change of indicated ISGs and pro inflammatory cytokines were shown.

### ELISA

Serum insulin, GADA, IAA, ICA and IFN-β were measured using Rat/mouse insulin ELISA Kit (Millipore, EZRMI-13K), Rat GAD-Ab ELISA Kit (Shanghai Jiang Lai, JL11155), Rat IAA ELISA Kit (Shanghai Jiang Lai, JL10558), Rat ICA ELISA Kit (Shanghai Jiang Lai, JL10634) and Rat Interferon Beta (IFNβ) ELISA Kit (Shanghai Jiang Lai, JL20851), respectively. These assays were performed according to manufacturer’s instructions.

### Biochemical assays

Serum of WT or *Trex1*^−/−^ rats was collected and the levels of HbA1c, urea and creatinine in serum were analyzed by Beijing BJ•XinChuangYuan BIOTECH CO., LTD. (China). 24-h urine from each rat was collected with a metabolic cage. Urinary albumin and creatinine were analyzed by Beijing BJ•XinChuangYuan BIOTECH CO., LTD. (China).

### Cataract grading

Using a slit lamp, the dilated pupils were examined regularly. According to the turbidity of lens, cataracts were graded into 6 stages by two experienced ophthalmologists. Stage 0: The lens is transparent. Stage 1: Cataract with a slight degree of punctate opacification. Stage 2: Cataract with an enlarged punctate opacification, which occupy 1/4 of the lens. Stage 3: Wheel-like turbidity over two quadrants. Stage 4: Wheel-like turbidity over three quadrants. Stage 5: The entire lens is turbid.

### Statistical analysis

The statistical analysis and sample sizes are descripted in the figure legends. The statistics were performed using GraphPad Prism (GraphPad Software). Briefly, the Shapiro–Wilk test and F test was employed to test normality and homogeneity of variance, respectively. For data of two groups that meet the normal distribution, unpaired t-test or Welch’s t test was applied; While the data do not meet the normal distribution, we used Mann–Whitney test. Multiple comparisons were carried out by Two-way ANOVA, and Log-rank (Mantel-Cox) test was used in the survival analysis. *P* values are shown in the figures. Data are shown as mean ± SEM.

## Results

### *Trex1*-deficient rats develop spontaneous diabetes

We generated *Trex1*-deficient Sprague Dawley (SD) rats by deleting 122 base pairs in exon 2 of *Trex1* gene using CRISPR-Cas9 (Additional file 2: Fig. S1A–C). Unexpectedly, we noticed that the *Trex1*^*−/−*^ rats displayed diabetic-like symptoms, including polydipsia (increased thirst) and polyphagia (increased appetite) (Additional file 2: Fig. S1D). To further investigate this phenomenon, we performed a long-term monitoring of fasting blood glucose in wild-type (WT) and *Trex1*^*−/−*^ rats, starting from 4 weeks of age, with a frequency of once a month, lasting for 1 years. We found that *Trex1*^*−/−*^ rats exhibited evidently elevated blood glucose compared to WT rats (Fig. 1A). According to the diagnostic criteria for diabetes, we considered the blood glucose level over 13.8 mmol/L for two consecutive measurements as diabetic [22–24]. The prevalence of diabetes in *Trex1*^*−/−*^ rats progressively increased with age, and approximately 70% of both male and female rats developed diabetes by 48 weeks of age (Fig. 1B, Additional file 2: Fig. S1E). Additionally, we observed that *Trex1*^*−/−*^ rats had a higher hemoglobin A1c (HbA1c) than WT rats, and the glucose tolerance in *Trex1*^*−/−*^ rats was also impaired, supporting that *Trex1*^*−/−*^ rats are deficient in glycemic control (Fig. 1C–E). Furthermore, compared to WT rats, the lifespan of *Trex1*^*−/−*^ rats was shortened after onset of diabetes (Fig. 1F). Thus, these data suggest that *Trex1*^*−/−*^ rats spontaneously develop diabetes.

Streptozotocin (STZ) is widely utilized in chemically induced diabetes model for preclinical diabetes research [25–27]. We next treated WT and *Trex1*^*−/−*^ rats with a single low-dose STZ (30 mg/kg), a dose of which is insufficient to induce diabetes in WT rats. Following STZ treatment, *Trex1*^*−/−*^ rats exhibited an earlier onset and higher incidence of diabetes (Fig. 1,G, H), indicating the increased susceptibility to STZ-induced diabetes in *Trex1*^*−/−*^ rats.

### *Trex1*-deficient rats develop type 1 diabetes

**Fig. 2:**
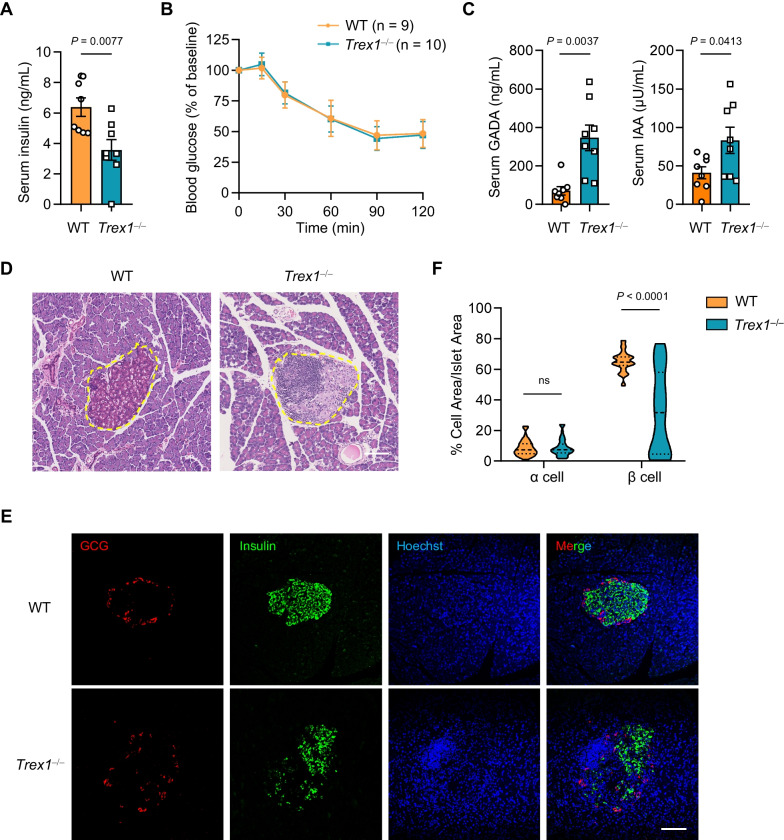
*Trex1*-deficient rats develop Type 1 diabetes. **A** Serum insulin levels were measured in 8-week-old WT (n = 8) and *Trex1*^*−/−*^ (n = 8) rats after fasting for 4 h. **B** Blood glucose levels during an intraperitoneal insulin tolerance test were monitored in WT (n = 9) and *Trex1*^*−/−*^ (n = 10) rats at 16 weeks of age. **C** Serum GADA and IAA levels were measured by Elisa in 16-week-old WT (n = 8) and *Trex1*^*−/−*^ (n = 8) rats. **D** Representative H&E staining images of pancreatic section from WT and *Trex1*^*−/−*^ rats. Yellow circles indicate islets. Scale bar, 100 μm. **E** Representative fluorescence images of pancreatic sections from WT and *Trex1*^*−/−*^ rats stained with insulin (green) and glucagon (red) antibodies, indicating β cells and α cells respectively. The cell nuclei were stained with Hoechst (blue). Scale bar, 100 μm. **F** Percentage of β cells and α cells in islets from WT (n = 3) and *Trex1*^*−/−*^ (n = 3) rats. 15 islets in each of the rats were analyzed. All data are represented as mean ± SEM; Each dot represents one independent biological replicate; Unpaired *t* test in **A**, (**C** IAA); Welch’s *t* test in (**C** GADA); Two-way ANOVA with Sidak correction in **F**

To elucidate the role of TREX1 in spontaneous development of diabetes, we first examined the type of diabetes that *Trex1*^*−/−*^ developed. Generally, T1D results from destruction of pancreatic β-cells, which causes defective secretion of insulin, while Type 2 diabetes arises from insulin resistance [28–30]. We then measured the fasting insulin in serum, and found that *Trex1*^*−/−*^ rats exhibited apparently deficient, even absent, insulin secretion (Fig. 2A). Meanwhile, insulin tolerance test showed no significant difference in blood glucose changes between WT and *Trex1*^*−/−*^ rats (Fig. 2B). The presence of autoantibodies in serum is an important indicator of T1D, including glutamic acid decarboxylase autoantibodies (GADA), insulin autoantibodies (IAA) and islet cell antibodies (ICA). We found that serum levels of the above-mentioned autoantibodies were significantly elevated in *Trex1*^*−/−*^ rats when compared to those in WT rats (Fig. 2C, Additional file 3: Fig. S2A). These results demonstrated that *Trex1*^*−/−*^ rats manifested T1D-like characteristics. We next verified the morphology and integrity of islets in *Trex1*^*−/−*^ rats. Hematoxylin and eosin (H&E) staining was performed and showed that the pancreatic islets were damaged in *Trex1*^*−/−*^ rats, accompanied by infiltration of immune cells (Fig. 2D). Furthermore, we observed remarkable loss of β-cells in pancreatic islets of *Trex1*^*−/−*^ rats, while their α-cells had little difference compared to WT rats (Fig. 2E, F). Taken together, these data suggest that *Trex1*-deficient rats develop T1D.

### *Trex1*-deficient rats exhibit insulitis and infiltration of immune cells

**Fig. 3:**
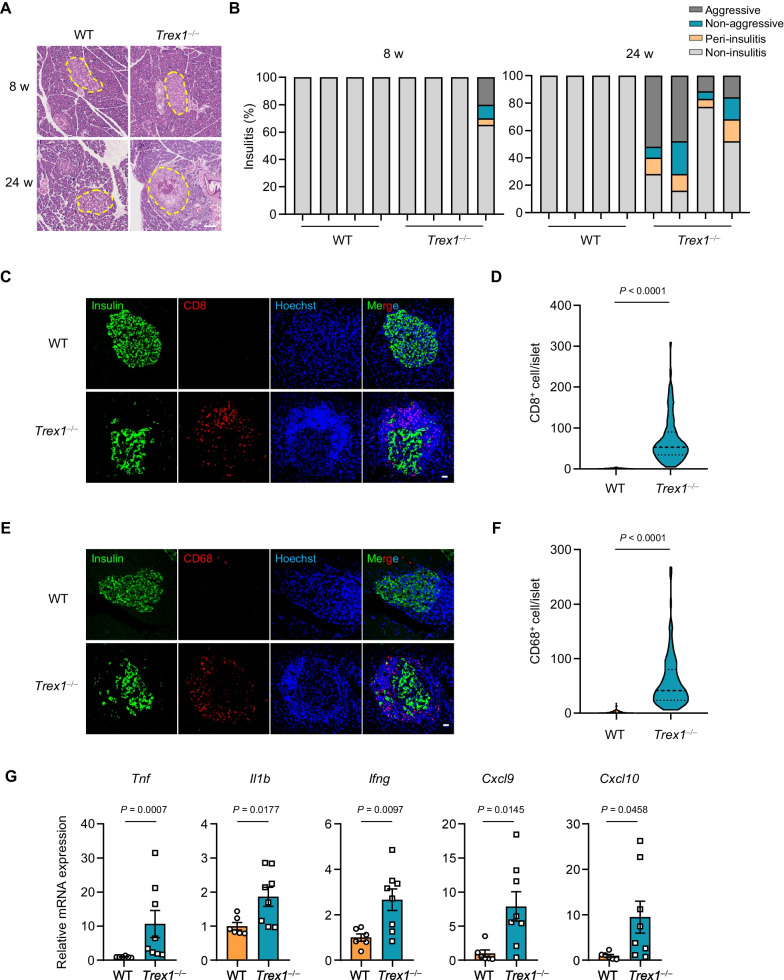
*Trex1*-deficient rats exhibit insulitis and infiltration of immune cells. **A** Representative H&E staining images of pancreatic sections from WT and *Trex1*^*−/−*^ rats at 8-week-old and 24-week-old. Yellow circles indicate islets. Scale bar, 100 μm. **B** Insulitis scoring was assessed on H&E-stained pancreatic sections from WT (n = 4) or *Trex1*^*−/−*^ (n = 4) rats at 8 or 24 weeks of age. 20–30 islets in each of the rats were analyzed. **C** Representative fluorescence images of pancreatic sections from WT or *Trex1*^*−/−*^ rats co-staining by insulin (green) and CD8 (red) antibodies. The cell nuclei were stained with Hoechst (blue). Scale bar, 20 μm. **D** Numbers of CD8^+^ cells in each islet from WT (n = 5) or *Trex1*^*−/−*^ (n = 5) rats were quantified. 20 islets in each of the rats were analyzed. **E** Representative fluorescence images of pancreatic sections from WT or *Trex1*^*−/−*^ rats co-stained by insulin (green) and CD68 (red) antibodies. The cell nuclei were stained with Hoechst (blue). Scale bar, 20 μm. **F** Numbers of CD68^+^ cells in each islet from WT (n = 5) or *Trex1*^*−/−*^ (n = 5) rats were quantified. 20 islets in each of the rats were analyzed. **G** The mRNA levels of indicated genes were analyzed by qPCR in pancreas from WT (n = 6) or *Trex1*^*−/−*^ (n = 8) rats. Normalized by the housekeeping gene *Gapdh*. All data are represented as mean ± SEM; Each column represents one rat in **B**; Each dot represents one islet in **D**, **F** or independent biological replicate in **G**; Mann Whitney test in **D**, **F** and (**G** *Tnf*); Welch’s *t* test in (**G** *Il1b, Ifng, Cxcl9, Cxcl10*)

The development of T1D is usually associated with insulitis triggered by infiltrated immune cells and local inflammation [31–33]. As the above H&E data suggested that pancreatic islets were infiltrated by immune cells in *Trex1*^*−/−*^ rats, we further confirmed this phenomenon using pancreatic sections adopted from both 8- and 24-week WT and *Trex1*^*−/−*^ rats. Histological analysis revealed that *Trex1*^*−/−*^ rats appeared varying degrees of insulitis with age, and the incidence and severity of which showed a notable increase at 24 weeks of age (Fig. 3A, B and Additional file 3: Fig. S2B). We also observed considerable infiltration of CD8-positive T-cells and CD68-positive macrophages in pancreatic islets of *Trex1*^*−/−*^ rats, compared to those in WT rats (Fig. 3C–F). Besides, we detected up-regulated expression of pro-inflammatory cytokines, such as *Tnf*, *Il1b*, *Ifng*, *Cxcl9* and *Cxcl10* in the pancreas of *Trex1*^*−/−*^ rats (Fig. 3G). Thus, these data suggest that deficiency of *Trex1* induces infiltration of immune cells into pancreatic islets which may create an inflammatory environment.

### *Trex1* deficiency results in accumulation of ssDNA and elevated production of IFN-β in pancreas

**Fig. 4:**
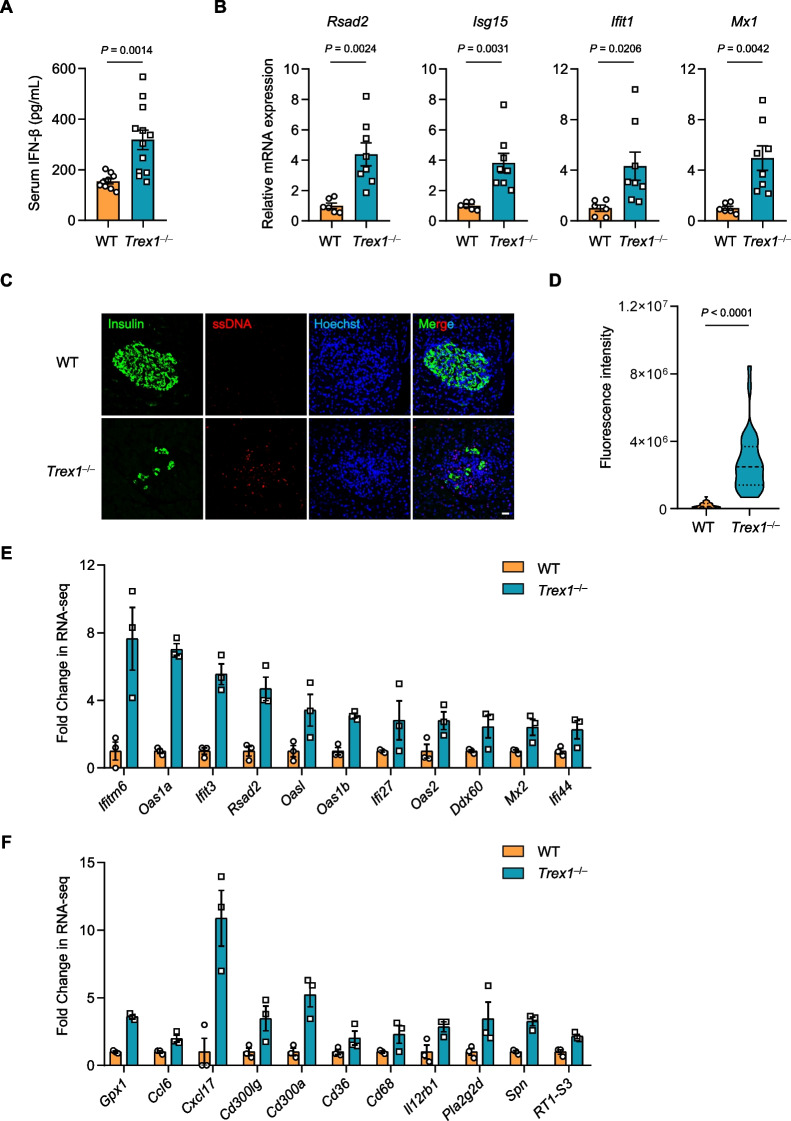
*Trex1* deficiency results in accumulation of ssDNA and elevated production of IFN-β in pancreas. **A** Serum from WT (n = 9) or *Trex1*^*−/−*^ (n = 12) rats were obtained for ELISA analysis of IFN-β concentration. **B** The mRNA levels of *Rsad2*, *Isg15*, *Ifit1*, *Mx1* in pancreas from WT (n = 6) and *Trex1*^*−/−*^ (n = 8) rats were measured by qPCR. Normalized by the housekeeping gene *Gapdh*. **C** Representative fluorescence images of the pancreas from WT or *Trex1*^*−/−*^ rats stained with antibodies recognizing ssDNA (red) and insulin (green). Nuclei were stained with Hoechst (blue). Scale bar, 20 μm. **D** Fluorescence scoring of ssDNA^+^ islets cell from WT or *Trex1*^*−/−*^ rats were quantified. (n = 3 per group). 10 islets in each of the rats were analyzed. **E** Fold change of indicated ISGs transcripts from RNA-seq data in the pancreas of WT or *Trex1*^*−/−*^ rats. **F** Fold change of indicated pro-inflammatory cytokines genes transcripts from RNA-seq data in the pancreas of WT or *Trex1*^*−/−*^ rats. All data are represented as mean ± SEM; Each dot represents independent biological replicate; Welch’s* t* test in **A**, **B**; Mann Whitney test in **D**

Previous works have revealed that TREX1 deficiency is implicated in the development of several autoimmune diseases, mechanistically through the sustained activation of type I IFN pathway induced by aberrant accumulation of DNA [17, 34, 35]. We therefore speculated that the insulitis observed in *Trex1*^*−/−*^ rats might be attributed to the aberrant activation of IFN signaling in pancreatic islets. To prove this, we first detect the production of type I IFN in *Trex1*^*−/−*^ rats, and observed an obvious increase of IFN-β in the serum of *Trex1*^*−/−*^ rats (Fig. 4A). Interferon-stimulated genes (ISGs), including *Rsad2*, *Isg15*, *Ifit1* and *Mx1*, also showed enhanced expression in the pancreas obtained from *Trex1*^*−/−*^ rats (Fig. 4B). Moreover, immuno-staining of ssDNA with β-cell marker revealed substantially accumulated ssDNA around the islet β cells of *Trex1*^*−/−*^ rats, indicating that the activation of IFN signaling in *Trex1*^*−/−*^ rats is possibly induced by the accumulation of ssDNA in pancreatic islets (Fig. 4C, D). We next performed an RNA-seq using the pancreas tissue of WT and *Trex1*^*−/−*^ rats, and identified 598 differentially expressed genes (335 up-regulated and 263 down-regulated, fold change > 2, *P* value < 0.05) (Additional file 4: Fig. S3A, B). Among the down-regulated genes, KEGG analysis uncovered notable enrichment of terms associated with insulin secretion (Additional file 4: Fig. S3C), supporting the T1D phenotype observed in *Trex1*^*−/−*^ rats (Fig. 2). We also analyzed the up-regulated differentially expressed genes in RNA-seq data. Compared to WT rats, we found elevated expression of a number of ISGs (Fig. 4E), as well as a series of pro-inflammatory cytokines and chemokines in *Trex1*^*−/−*^ rats (Fig. 4F). These data together suggest that the pancreas of *Trex1*^*−/−*^ rats exhibits IFN-related gene signature, which potentially contributes to the development of diabetes.

### *Trex1*-deficient rats develop diabetic cataracts and diabetic nephropathy

**Fig. 5:**
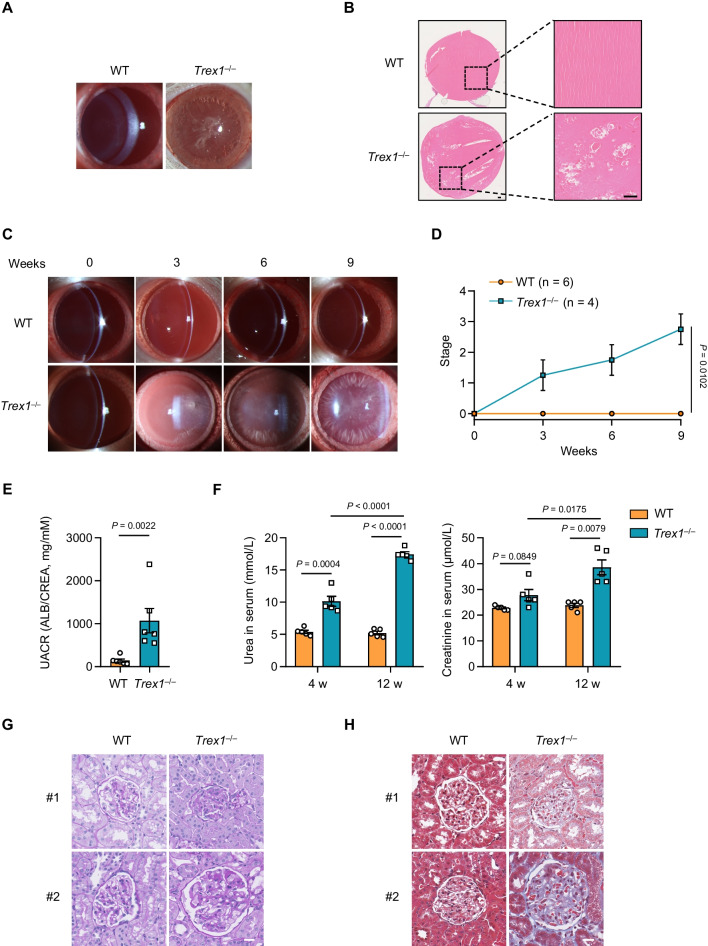
*Trex1*-deficient rats develop diabetic cataracts and diabetic nephropathy. **A** Representative slit-lamp photographs of lens from WT or *Trex1*^*−/−*^ rats. **B** Representative H&E images showing lens sections from WT or *Trex1*^*−/−*^ rats. Scale bars, 100 μm. **C** Representative slit-lamp photographs of lens from different groups of animals at 0, 3, 6 and 9 weeks after the onset of diabetes in *Trex1*^*−/−*^ rats. **D** The progression of diabetic cataracts in WT (n = 6) or *Trex1*^*−/−*^ (n = 4) rats was quantified. **E** The UACR levels were calculated by the ratio of urinary albumin and creatinine in *Trex1*^*−/−*^ rats at 8 and 12 weeks after the onset of diabetes (n = 6 per group). **F** The urea and creatinine levels in serum were quantified at 4 and 12 weeks after the onset of diabetes in *Trex1*^*−/−*^ rats (n = 5 in each condition). **G**, **H** Representative images of kidney sections from WT or *Trex1*^*−/−*^ rats stained by periodic acid–Schiff (PAS) **G** or Masson’s trichrome **H**. Scale bars, 20 μm. All data are represented as mean ± SEM; Each dot represents independent biological replicate; Repeated measure ANOVA with Sidak post hoc test in **D**; Mann Whitney test in **E**, (**F** creatinine 12 w); Unpaired *t*-test in (**F** urea, **F** creatinine 4 w and 12 w); Welch’s* t* test in (**F** creatinine 4 w)

Diabetic patients are usually burdened with different complications, such as diabetic cataracts, peripheral neuropathy and diabetic nephropathy [36, 37]. We therefore determined whether *Trex1*^*−/−*^ rats developed similar complications in addition to diabetic phenotype. We noticed that after onset of diabetes, lens of the *Trex1*^*−/−*^ rats developed a cloudy and white appearance, resembling the manifestation of diabetic cataracts (DC) in human (Fig. 5A). Histological analysis revealed that the cytoarchitecture of lens in *Trex1*^*−/−*^ rats showed disordered arrangement and the cortical fiber cells displayed a number of vacuoles (Fig. 5B). We next performed the slit-lamp examination to assess the progression of diabetic cataracts in *Trex1*^*−/−*^ rats. The turbidity levels of lens were graded into six stages by professional ophthalmologists (Additional file 5: Fig. S4A). Our data showed that *Trex1*^*−/−*^ rats gradually developed cataract since the onset of diabetes, with mature cataract formation by 9 weeks after diagnosis (Fig. 5C, D). These findings strongly suggest that *Trex1*^*−/−*^ rats develop diabetic cataracts after onset of diabetes.

Diabetic nephropathy, one of the most common chronic diabetic complications, is characterized by both functional and structural changes, including albuminuria, glomerulosclerosis, mesangial expansion and tubulointerstitial fibrosis [38, 39]. We observed elevated levels of albuminuria, creatinine and urea in *Trex1*^*−/−*^ rats after onset of diabetes, compared to those of WT rats (Fig. 5E, F and Additional file 5: Fig. S4B). We also found the levels of creatinine and urea in serum of *Trex1*^*−/−*^ rats increased along time after onset of diabetes (Fig. 5F), suggesting the occurrence and progression of renal damage along disease course. Additionally, histopathological changes were analyzed by periodic acid-Schiff (PAS) staining and Masson trichrome staining. Thickening glomerular basement membrane (GBM), expansive mesangial matrix and renal fibrosis were observed in the kidney of diabetic *Trex1*^*−/−*^ rats (Fig. 5G, H). Moreover, the *Trex1*^*−/−*^ rats also displayed obvious pathological changes in the tubulointerstitial, such as the loss of tubular cells and tubulointerstitial fibrosis (Additional file 5: Fig. S4C). Further examination uncovered enhanced infiltration of CD68-positive macrophages in the renal tissues of diabetic *Trex1*^*−/−*^ rats (Additional file 5: Fig. S4D, E), supporting the conclusion that *Trex1*^*−/−*^ rats develop diabetic nephropathy after onset of diabetes.

## Discussion

TREX1, as a DNA-degrading enzyme, is closely associated with DNA-sensing mediated innate immune signaling and development of several autoimmune diseases. In this study, we discovered that *Trex1*^*−/−*^ rats exhibit high levels of blood glucose, together with insufficient insulin secretion, indicating these rats spontaneously develop T1D. *Trex1*^*−/−*^ rats also gradually develop diabetic complications, such as cataracts and nephropathy, after onset of diabetes. Our study thus provides a potential diabetes model which may contribute to exploring the pathogenesis of T1D.

Diabetic animal models are of great significance for investigations of pathogenic mechanisms and development of therapeutic strategies. Although several animal models, including NOD mice, BB rats and STZ-induced diabetic model, have been widely employed in diabetes research, there still remain some limitations. For example, BB rats develop lymphopenia in addition to diabetic phenotype, which differs from symptoms of diabetes patients [40–42]. NOD mice exhibit notable differences in diabetes incidence between males and females [8, 43, 44]. In the present work, we demonstrated that *Trex1*^*−/−*^ rats spontaneously develop diabetes starting from 4 weeks of age after birth, with a diabetes incidence of up to 70% by 1 year and no significant differences in diabetes incidence between males and females. *Trex1*^*−/−*^ rats also develop diabetic nephropathy and diabetic cataract after the onset of diabetes, which resembles the complications as diabetes patients. The development of these complications in *Trex1*^−/−^ rats may result from prolonged exposure of hyperglycemia, while the possibility that *Trex1* deficiency leads to cell death and functional decline in eye and kidney, thereby contributing to the pathogenesis of nephropathy and cataracts, still could not be excluded.

Growing evidences have indicated an important role for type I IFN in the development of T1D [45–47]. It was reported that a remarkable type I IFN gene signature was observed in the islets and peripheral blood of individuals with T1D, which appears before the emergence of autoantibodies [45, 48]. By targeting type I IFN-mediated signaling pathway, such as type I IFN receptor, the occurrence of T1D could be delayed [49]. In addition to this, viral infection is one of the environmental risk factors of T1D, which is usually featured by elevated production of IFN. Our study found an evident accumulation of ssDNA, together with an obvious IFN-related gene signature, in pancreata of *Trex1*^*−/−*^ rats. As previous studies suggested that *TREX1* deficiency leads to accumulation of intracellular DNA, thereby activating cGAS-mediated DNA-sensing pathway and facilitating production of type I IFN, our study thus provides new evidence for the connection between type I IFN and T1D.

Aberrant activation of cGAS has been implicated in the development of autoimmune diseases such as AGS and SLE, and targeting cGAS is becoming a promising strategy for treatment of such diseases. Further studies are needed to determine whether cGAS activation interprets the role of *Trex1* deficiency in T1D. And examinations regarding polymorphisms of *TREX1* gene and activation of cGAS in diabetic patients are also needed to support their contribution in the pathogenesis of T1D.

## Conclusions

In summary, we found that *Trex1* deficiency leads to spontaneous development of T1D in rats, accompanied by complications such as diabetic nephropathy and cataract. Local inflammation in pancreas of *Trex1*^*−/−*^ rats may be caused by excessive production of proinflammatory cytokines, including type I IFN, which eventually leads to the decline of pancreatic β cells. This study may provide a new animal model for T1D research and new insights into the pathogenesis of T1D.

### Supplementary Information

**Additional file 1: Table S1.** List of qPCR primer sequences.

**Additional file 2: Fig. S1.** *Trex1*-deficient rats display diabetic-like symptoms. (A) Schematic drawing of *Trex1 *gene of rat, red arrowhead indicates the sgRNA target sites. (B) The *Trex1* gene of WT or *Trex1*^−/−^ rats was detected by PCR. The smaller PCR products amplified in *Trex1*^−/−^ rats reflect deletions with CRISPR-Cas9 system. (C) The mRNA expression of *Trex1* in indicated tissues from WT or *Trex1*^−/−^ rats (n=3 per group) was analyzed by qPCR. (D) The water and food intake in WT (n=11) and *Trex1*^−/−^ (n=13) rats was monitored for 24 hours. (E) Incidence of diabetes in *Trex1*^−/−^ female (n=26) and *Trex1*^−/−^ male (n=20) rats from 4 weeks postnatal until 48 weeks. All data are represented as mean ± SEM; Each dot represents independent biological replicate; Unpaired *t*-test in (C); Mann Whitney test in (D, Food intake); Welch’s *t* test in (D, Fluid intake); Log-rank (Mantel-Cox) test in (E).

**Additional file 3: Fig. S2.** Serum ICA levels and insulitis scoring. (A) Serum ICA levels were measured by Elisa in WT (n=8) and *Trex1*^−/−^ (n=8) rats. (B) Representative H&E images of the stages of insulitis. Scale bar, 100 μm. All data are represented as mean ± SEM; Welch’s *t* test in (A).

**Additional file 4: Fig. S3.** RNA-seq analysis of the pancreas from WT or *Trex1*^−/−^ rats. (A) The RNA integrity number values of RNA-seq samples. (B) Volcano plots of significantly differentially expressed genes of the RNA-seq data from pancreas of WT and *Trex1*^−/−^ rats (n=3 per group). Red, down-regulated; Green, up-regulated. (C) KEGG analysis of down-regulated genes (*Trex1*^−/−^ vs. WT) in pancreas RNA-seq data set.

**Additional file 5: Fig. S4.** *Trex1* deficiency leads to the development of diabetic cataract and diabetic nephropathy in rats. (A) Representative slit-lamp photos of the grading of diabetic cataract. (B) The UACR levels were calculated by the ratio of urinary albumin and creatinine in *Trex1*^−/−^ rats at 8 or 12 weeks after the onset of diabetes (n=3 in each condition). (C) Representative images of kidney tubules from WT or *Trex1*^−/−^ rats stained by PAS or Masson’s trichrome. Scale bar, 40 μm. (D) Representative fluorescence images of kidney sections from WT or *Trex1*^−/−^ rats stained with CD68 (red) antibody. The cell nuclei were stained with Hoechst (blue). Scale bar, 20 μm. (E) The number of CD68^+^ cells in kidney from WT (n=3) or *Trex1*^−/−^ (n=3) rats were quantified. 10 glomeruli were analyzed in each of the rats. Data are represented as mean ± SEM; Each dot represents independent biological replicate; Unpaired *t*-test in (B UACR 8 w, UACR 8 w and 12 w); Welch’s *t *test in (B UACR 12 w, E).

## Data Availability

All RNA sequencing files were deposited in the NCBI Sequence Read Archive under the BioProject number PRJNA1027006. qPCR primers used in this study are included in Additional file 1: Table S1. Other data presented in this study are available from the corresponding authors on reasonable request.

## Abbreviations

TREX1: Three prime repair exonuclease 1
SD: Sprague Dawley
T1D: Type 1 diabetes
IFN: Interferon
BB: BioBreeding
cGAS: Cyclic GMP–AMP synthase
AGS: Aicardi–Goutières syndrome
SLE: Systemic lupus erythematosus
FCL: Familial chilblain lupus
RVCL: Retinal vasculopathy with cerebral leukodystrophy
STZ: Streptozotocin
GTT: Glucose tolerance test
ITT: Insulin tolerance test
qPCR: Quantitative PCR
DEGs: Differentially expressed genes
GADA: Glutamic acid decarboxylase autoantibodies
IAA: Insulin autoantibodies
ICA: Islet cell antibodies
WT: Wild-type
HbA1c: Hemoglobin A1c
H&E: Hematoxylin and eosin
ISG: Interferon-stimulated gene
DC: Diabetic cataracts
