# Supplementary figures and images for: Deficiency of Trex1 leads to spontaneous development of type 1 diabetes
Source: Nutr Metab (Lond). 2024 Jan 2;21:2. doi: 10.1186/s12986-023-00777-6 (PMC10763031; doi:10.1186/s12986-023-00777-6)

**A**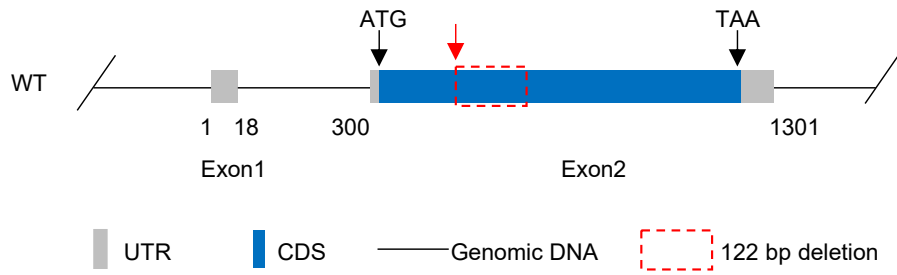**B**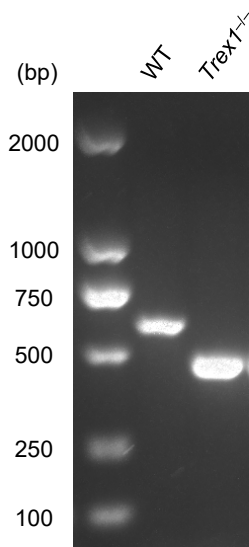**C**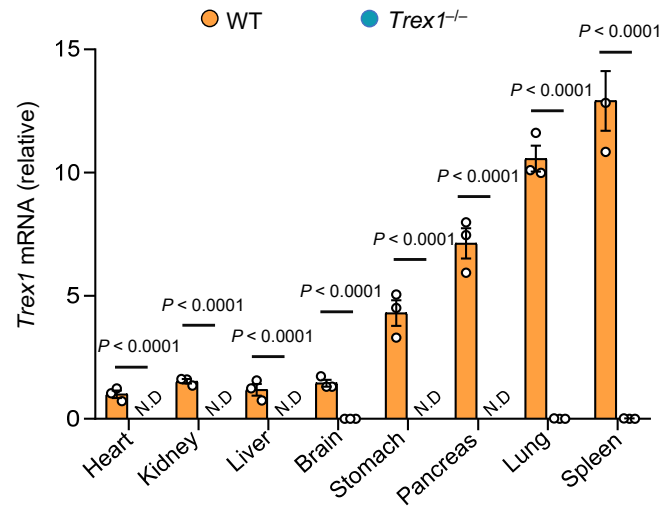**D**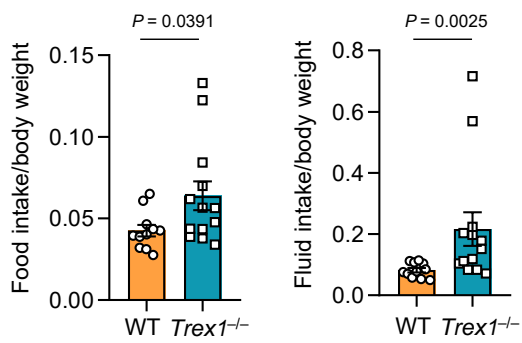**E**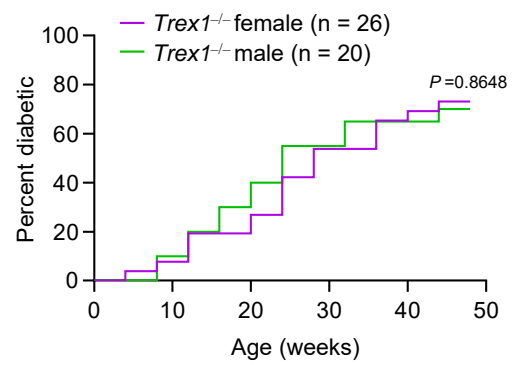

Supplement: Supplementary file 2 — Additional file 2: Fig. S1. Trex1-deficient rats display diabetic-like symptoms. (A) Schematic drawing of Trex1 gene of rat, red arrowhead indicates the sgRNA target sites. (B) The Trex1 gene of WT or Trex1−/− rats was detected by PCR. The smaller PCR products amplified in Trex1−/− rats reflect deletions with CRISPR-Cas9 system. (C) The mRNA expression of Trex1 in indicated tissues from WT or Trex1−/− rats (n=3 per group) was analyzed by qPCR. (D) The water and food intake in WT (n=11) and Trex1−/− (n=13) rats was monitored for 24 hours. (E) Incidence of diabetes in Trex1−/− female (n=26) and Trex1−/− male (n=20) rats from 4 weeks postnatal until 48 weeks. All data are represented as mean ± SEM; Each dot represents independent biological replicate; Unpaired t-test in (C); Mann Whitney test in (D, Food intake); Welch’s t test in (D, Fluid intake); Log-rank (Mantel-Cox) test in (E). [file 12986_2023_777_MOESM2_ESM.pdf]

**A**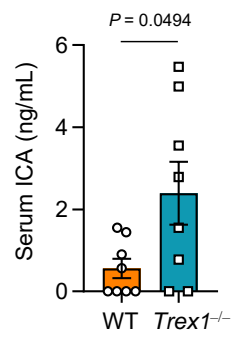**B**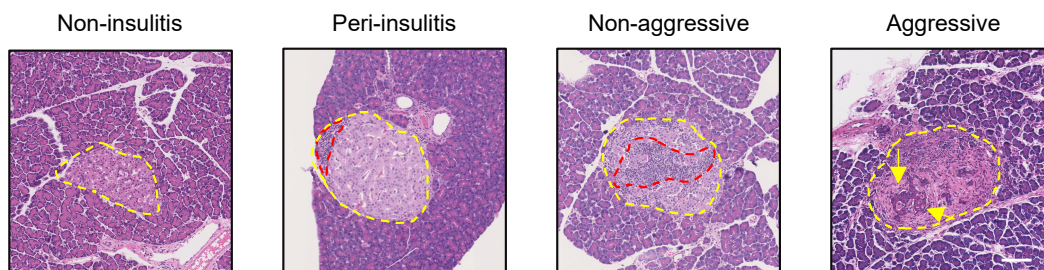

Supplement: Supplementary file 3 — Additional file 3: Fig. S2. Serum ICA levels and insulitis scoring. (A) Serum ICA levels were measured by Elisa in WT (n=8) and Trex1−/− (n=8) rats. (B) Representative H&E images of the stages of insulitis. Scale bar, 100 μm. All data are represented as mean ± SEM; Welch’s t test in (A). [file 12986_2023_777_MOESM3_ESM.pdf]

**A**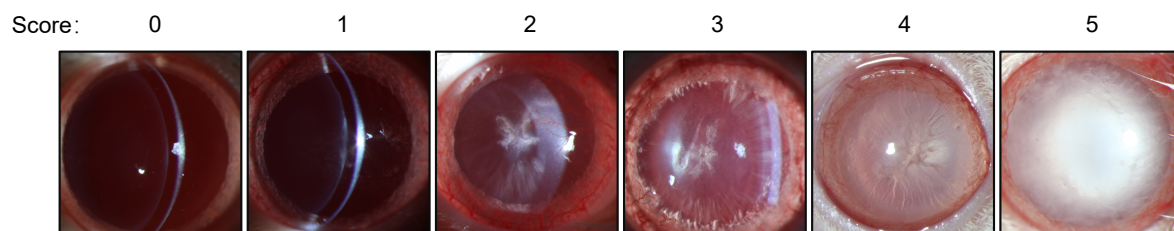**B**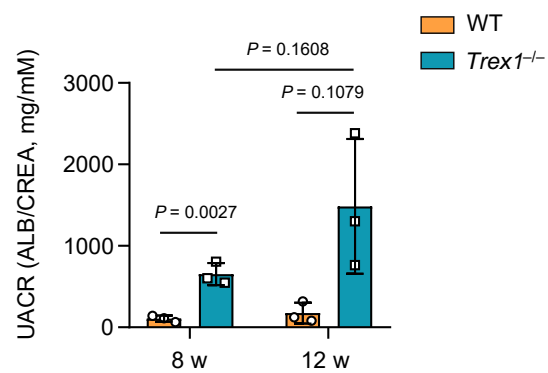**C**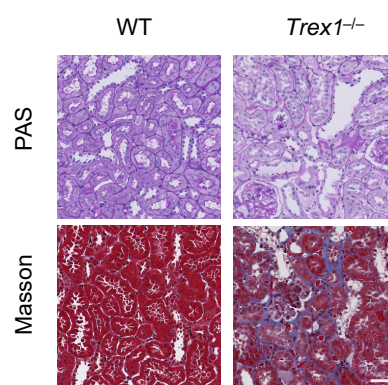**D**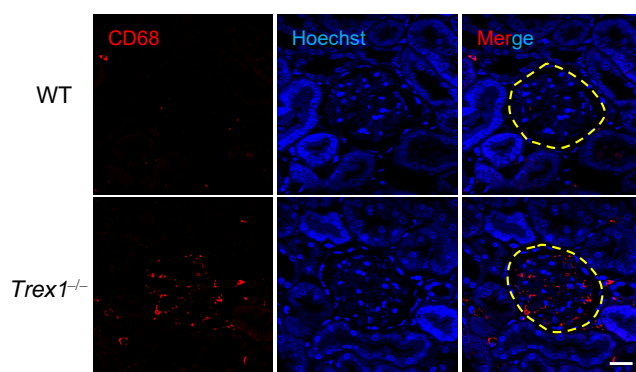**E**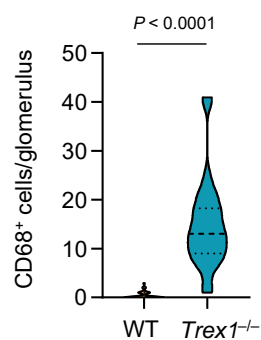

Supplement: Supplementary file 5 — Additional file 5: Fig. S4. Trex1 deficiency leads to the development of diabetic cataract and diabetic nephropathy in rats. (A) Representative slit-lamp photos of the grading of diabetic cataract. (B) The UACR levels were calculated by the ratio of urinary albumin and creatinine in Trex1−/− rats at 8 or 12 weeks after the onset of diabetes (n=3 in each condition). (C) Representative images of kidney tubules from WT or Trex1−/− rats stained by PAS or Masson’s trichrome. Scale bar, 40 μm. (D) Representative fluorescence images of kidney sections from WT or Trex1−/− rats stained with CD68 (red) antibody. The cell nuclei were stained with Hoechst (blue). Scale bar, 20 μm. (E) The number of CD68+ cells in kidney from WT (n=3) or Trex1−/− (n=3) rats were quantified. 10 glomeruli were analyzed in each of the rats. Data are represented as mean ± SEM; Each dot represents independent biological replicate; Unpaired t-test in (B UACR 8 w, UACR 8 w and 12 w); Welch’s t test in (B UACR 12 w, E). [file 12986_2023_777_MOESM5_ESM.pdf]
