# Supplementary material for: Deficiency of Trex1 leads to spontaneous development of type 1 diabetes
Source: Nutr Metab (Lond). 2024 Jan 2;21:2. doi: 10.1186/s12986-023-00777-6 (PMC10763031; doi:10.1186/s12986-023-00777-6)

A

The RNA Integrity Number values of RNA-seq samples

Sample	WT-1	WT-2	WT-3	*Trex1*$^{-/-}$-1	*Trex1*$^{-/-}$-2	*Trex1*$^{-/-}$-3
RIN	7.4	7.3	7.0	7.0	6.8	8.4

B

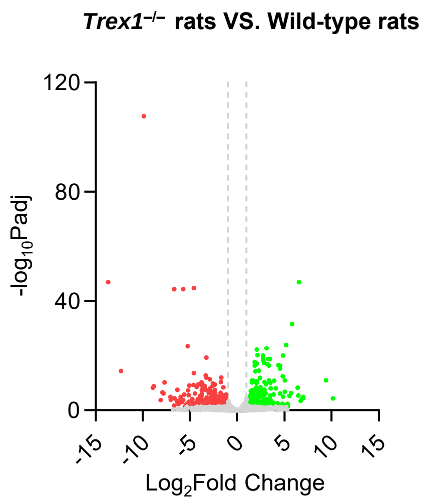

C

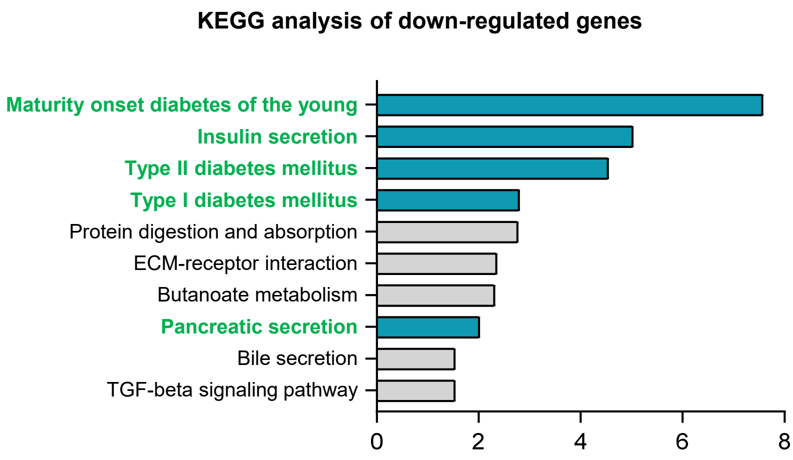

Supplement: Supplementary file 4 — Additional file 4: Fig. S3. RNA-seq analysis of the pancreas from WT or Trex1−/− rats. (A) The RNA integrity number values of RNA-seq samples. (B) Volcano plots of significantly differentially expressed genes of the RNA-seq data from pancreas of WT and Trex1−/− rats (n=3 per group). Red, down-regulated; Green, up-regulated. (C) KEGG analysis of down-regulated genes (Trex1−/− vs. WT) in pancreas RNA-seq data set. [file 12986_2023_777_MOESM4_ESM.pdf]
